# Marine Collagen/Apatite Composite Scaffolds Envisaging Hard Tissue Applications

**DOI:** 10.3390/md16080269

**Published:** 2018-08-03

**Authors:** Gabriela S. Diogo, Estefânia López-Senra, Rogério P. Pirraco, Raphael F. Canadas, Emanuel M. Fernandes, Julia Serra, Ricardo I. Pérez-Martín, Carmen G. Sotelo, Alexandra P. Marques, Pio González, Joana Moreira-Silva, Tiago H. Silva, Rui L. Reis

**Affiliations:** 1 3B’s Research Group—Biomaterials, Biodegradables and Biomimetics, University of Minho, Headquarters of the European Institute of Excellence on Tissue Engineering and Regenerative Medicine, AvePark, Parque de Ciência e Tecnologia, 4805-017 Barco, Guimarães, Portugal; gabriela.carlos@i3bs.uminho.pt (G.S.D.); rpirraco@i3bs.uminho.pt (R.P.P.); raphael.canadas@i3bs.uminho.pt (R.F.C.); efernandes@i3bs.uminho.pt (E.M.F.); apmarques@i3bs.uminho.pt (A.P.M.); moreirasilva.j@gmail.com (J.M.-S.); rgreis@i3bs.uminho.pt (R.L.R.); 2 ICVS/3B’s—PT Government Associate Laboratory, Braga/Guimarães, Portugal; 3 New Materials Group, Department of Applied Physics, Instituto de Investigación Sanitaria Galicia Sur IISGS, University of Vigo, Campus Lagoas-Marcosende, 36310 Vigo, Spain; estelopezsenra@gmail.com (E.L.S.); jserra@uvigo.es (J.S.); pglez@uvigo.es (P.G.); 4 Instituto de Investigaciones Marinas (CSIC), Eduardo Cabello 6, 36208 Vigo, Spain; ricardo@iim.csic.es (R.I.P.-M.); carmen@iim.csic.es (C.G.S.); 5 The Discoveries Centre for Regenerative and Precision Medicine, Headquarters at University of Minho, Avepark, 4805-017 Barco, Guimarães, Portugal

**Keywords:** marine biomaterials, composites, bone tissue engineering, collagen, calcium-phosphates, shark by-products

## Abstract

The high prevalence of bone defects has become a worldwide problem. Despite the significant amount of research on the subject, the available therapeutic solutions lack efficiency. Autografts, the most commonly used approaches to treat bone defects, have limitations such as donor site morbidity, pain and lack of donor site. Marine resources emerge as an attractive alternative to extract bioactive compounds for further use in bone tissue-engineering approaches. On one hand they can be isolated from by-products, at low cost, creating value from products that are considered waste for the fish transformation industry. One the other hand, religious constraints will be avoided. We isolated two marine origin materials, collagen from shark skin (*Prionace glauca*) and calcium phosphates from the teeth of two different shark species (*Prionace glauca* and *Isurus oxyrinchus*), and further proposed to mix them to produce 3D composite structures for hard tissue applications. Two crosslinking agents, 1-[3-(dimethylamino)propyl]-3-ethylcarbodiimide hydrochloride/N-Hydroxysuccinimide (EDC/NHS) and hexamethylene diisocyanate (HMDI), were tested to enhance the scaffolds’ properties, with EDC/NHS resulting in better properties. The characterization of the structures showed that the developed composites could support attachment and proliferation of osteoblast-like cells. A promising scaffold for the engineering of bone tissue is thus proposed, based on a strategy of marine by-products valorisation.

## 1. Introduction

The regeneration of bone defects represents a growing need and a worldwide concern [1]. Despite the high self-remodelling ability of bone tissue, severe damage can lead to a loss of this innate capacity. Bone grafts, including, autografts, allografts and xenografts are some of the approaches used to treat bone defects [2]. However, limitations such as donor site morbidity, pain and lack of donor site (autografts), and risk of disease transmission or rejection (allografts and xenografts) leave room for alternative approaches [3]. In that sense, tissue engineering and the development of temporary regenerative matrices that provide the 3D environment required for cell proliferation and function promoting tissue growth appears an attractive alternative. While polymers and ceramics have been largely explored to produce such matrices, reduced biocompatibility and risk of immunogenicity, respectively, due to synthetic and animal origin components are still observed. Marine resources emerge as an attractive alternative since they are free of mammalian diseases, or, to date, any other transmissible disease, and at the same time avoids religious constraints. Moreover, these marine-origin materials can be isolated from by-products, at low cost, creating value from products that are considered waste for the fish transformation industry, representing a great opportunity with huge potential [4,5,6].

For instance, fish skin and bones have a large amount of bioactive compounds with potential to be applied in health-related applications [5,7,8,9,10], with material properties depending on the raw material, source and extraction process [11]. Collagen and calcium phosphates represent the most abundant and explored compounds obtained from fish skin and bones, respectively.

Collagen isolated from salmon fish skin was used to produce 3D composites by a biomimetic mineralization process [8]. The high potential of these marine origin structures was demonstrated by the ability of the composites to promote osteogenic differentiation. Moreover, the suitability of marine origin collagen was also proven by other research groups. Fernandes-Silva and collaborators, also assessed the excellent properties of marine organisms as a collagen source through the use of shark collagen to produce hydrogels envisioning cartilage tissue regeneration [12].

Considering the excellent properties of calcium phosphates for hard tissue applications, recently the potential of bioapatites obtained from teeth of blue shark specie to promote osteogenic differentiation has been studied [13]. The calcium phosphates isolated from shark tooth are not only hydroxyapatite (HA), the most commonly used ceramic for hard tissue regeneration, but also HA enriched with fluorapatite Ca_5_(PO_4_)_3_F, unlike mammalian teeth. Moreover, some other elements, such as magnesium (Mg) and strontium (Sr), are present in their constitution [13,14]. These ions have been shown to stimulate osteoblastic activity by different mechanisms [15]. Mg ions, for instance, are involved in parathyroid hormone regulation, contributing to bone homeostasis [16,17,18,19] and fluorine has been demonstrated to promote bone formation by the stimulation of osteoblastic activity [20,21]. Thus, the presence of such ions in the mixture of the marine bioapatite particles (mBAp) and the incorporation of such particles into a type I marine collagen (mCol) matrix (the most abundant protein in hard tissues) can be a perfect combination to ensure good performance in hard tissue applications using marine origin resources. In that sense, in the present study collagen extracted from the skin of the blue shark (*Prionace glauca*) combined with bioceramic particles obtained from tooth of *Iurus oxyrinchus* and *Prionace glauca* were used to produce 3D structures aiming at hard tissue regeneration.

**Scheme 1:**
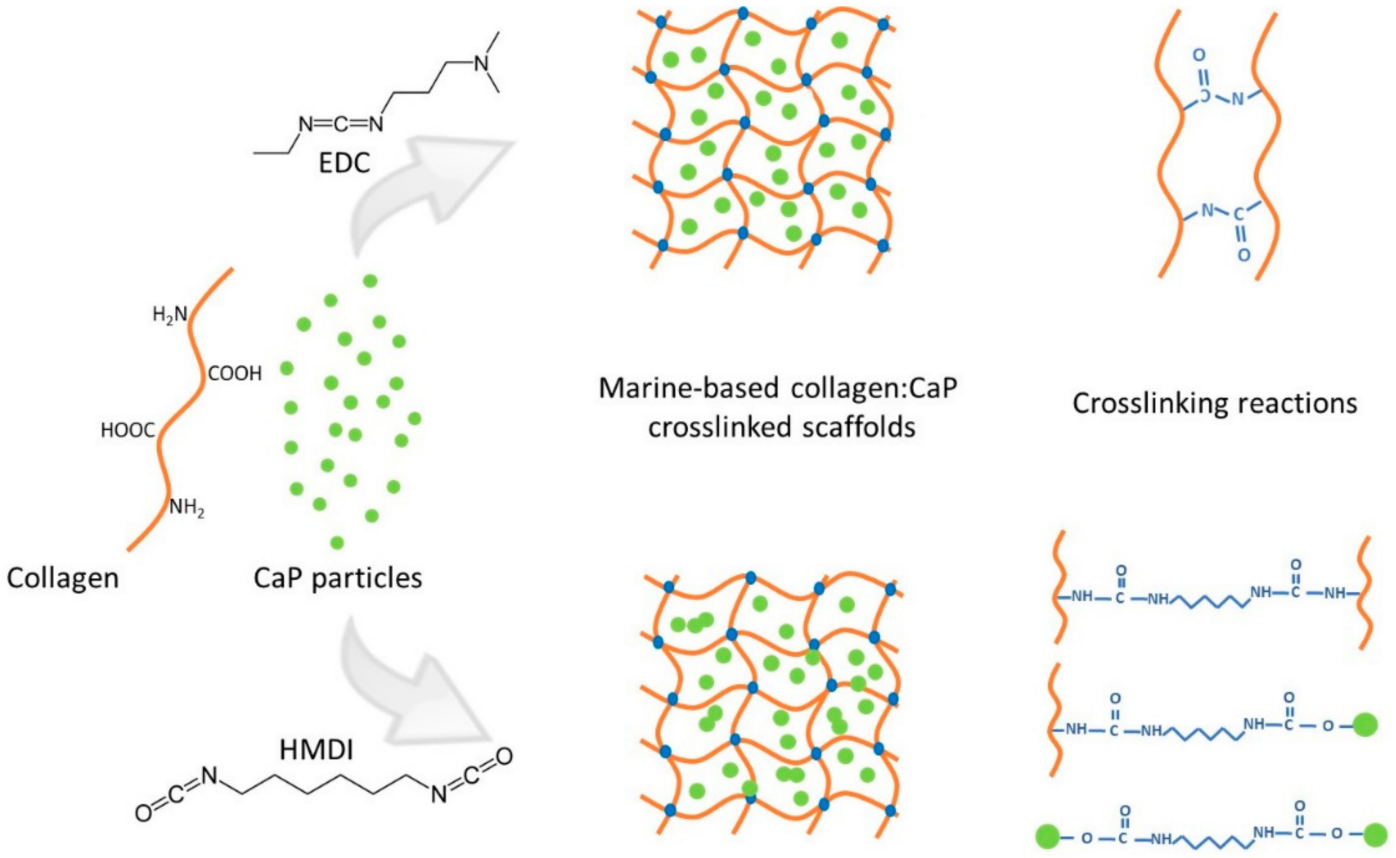
Schematic representation of crosslinking reactions for 1-[3-(dimethylamino)propyl]-3-ethylcarbodiimide hydrochloride (EDC) and hexamethylene diisocyanate (HMDI) crosslinking agents.

The physical combination of marine bioapatite particles with marine collagen used to develop the bone-like constructs was tested with two different crosslinking agents, 1-[3-(dimethylamino)propyl]-3-ethylcarbodiimide hydrochloride/N-Hydroxysuccinimide (EDC/NHS) or hexamethylene diisocyanate (HMDI), at different concentrations, to reinforce the constructs’ stability. The crosslinking approaches are illustrated in Scheme 1. The resulting scaffolds were characterized, addressing their morphological and mechanical properties, bioactivity upon immersion in simulated body fluid, cytotoxicity and performance towards bone tissue engineering after the culture of osteoblast-like cells.

## 2. Results and Discussion

### 2.1. Physicochemical Properties of Marine Bioapatite (mBAp)

The evaluation of marine bioapatite morphology derived from shark teeth was assessed in a previous study performed by López-Álvarez and collaborators [14,22].

By X-ray diffraction (XRD) it was possible to observe a combination of 65–70% apatitic phases (hydroxyapatite and fluorapatite) and 25–30% non-apatitic phases (tricalcium Bis (orthophosphate) and whitlockite) [23,24]. Morphological characterization by scanning electron microscopy (SEM) showed particles with a mean pore size of around 50 µm. Through inductively coupled plasma optical emission spectrometry (ICP-OES) and ion chromatography (Table 1), it was possible to observe that the main elements present in the marine bioapatite were calcium (Ca) and phosphates (P), followed by fluorine (F), sodium (Na) and magnesium (Mg). Other elements like strontium (Sr), aluminium (Al), and iron (Fe) were found in a lower concentration. Comparing this elemental composition with human bone content, we can see higher concentrations of Ca, P, F and Na ions for marine origin apatites [25]. These results are in accordance with some other previous studies performed with other marine organisms like cod fish [26].

### 2.2. Composite Scaffolds’ and Stability

**Figure 1:**
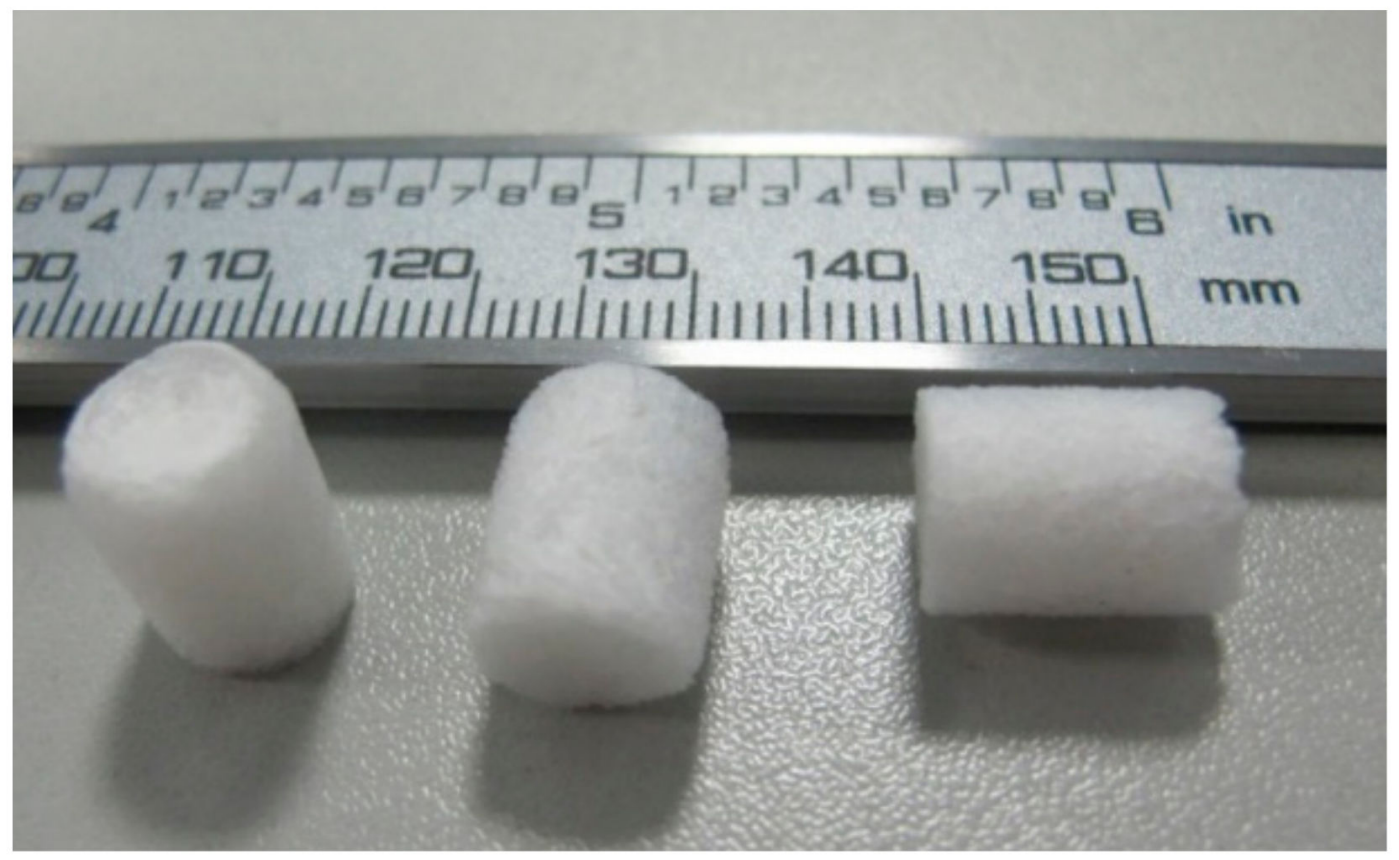
Representative composite scaffolds of mCol:mBAp.

Different composite scaffolds were produced by freeze-drying and crosslinking mixtures of shark skin collagen and shark teeth bioceramics. Figure 1 illustrates the structures obtained upon crosslinking with EDC/NHS or HMDI resulting in porous whitish sponges.

The structural stability of the scaffolds, as a measure of the effectiveness of crosslinking reaction and consecutively the success of the processing methodology, was assessed macroscopically after incubation in culture medium for 14 days. The results are summarized in Table 2. From the results, it is clear that there was an increase in the scaffolds’ stability in the presence of mBAp particles as expected. This result is in agreement with other reports that showed scaffolds with improved mechanical properties and slower degradation rate when compared with collagen alone, due to the incorporation of CaP [27]. In terms of crosslinker agents, we can observe similar stability for both 12.5%, 25% of EDC/NHS and 5% HMDI crosslinked scaffolds. Structures crosslinked with 1% HMDI were completely degraded 1 day after immersion in culture medium at 37 °C. This result suggests that 1% of HMDI may not be enough to efficiently crosslink the collagen in the scaffolds.

**Figure 2:**
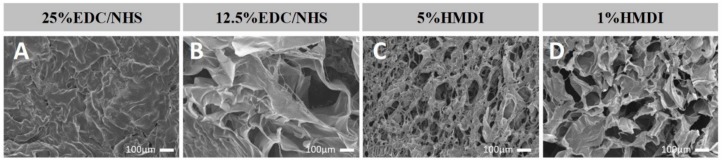
Representative scanning electron microscope (SEM) images of different mCol crosslinked scaffolds showing the crosslink effect over the microstructure. 25% (**A**) and 12.5% (**B**) EDC/NHS composite scaffolds´ and 5% (**C**) and 1% HMDI (**D**) composite scaffolds’.

In general, pore size and porosity decrease as the crosslink concentration increases. In the case of EDC/NHS, despite the stability results being similar for both concentrations a loss of the structural integrity for the 25% EDC/NHS formulation was macroscopically observed. These results were corroborated by SEM images (see Figure 2) which reveal shrinkage of collagen mesh for 25% EDC/NHS crosslinked scaffolds, with a consequent loss of porosity.

### 2.3. Composite Scaffolds’ Morphology

**Figure 3:**
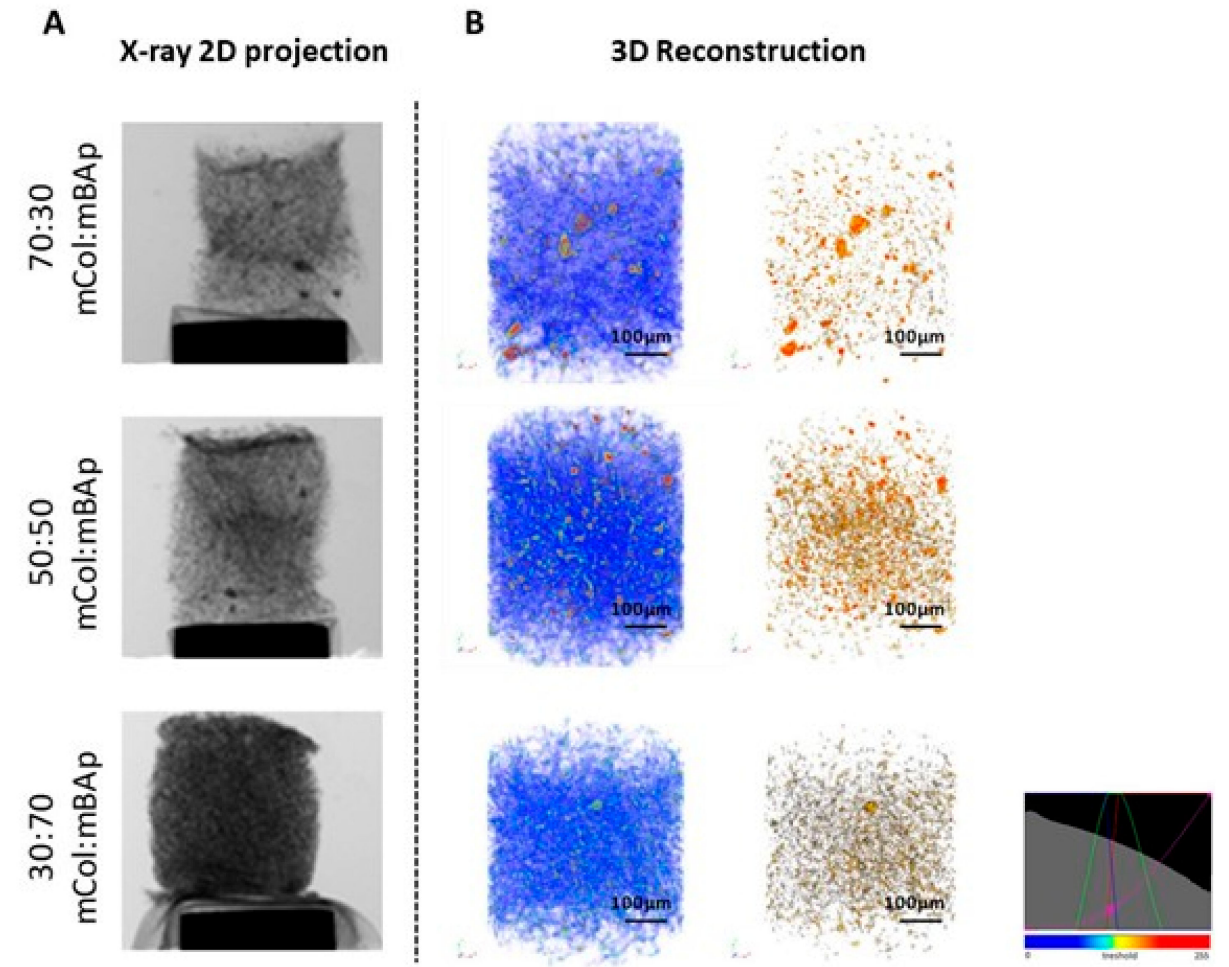
Representative images of 12.5% EDC/NHS crosslinked scaffolds obtained by microcomputed tomography (microCT). (**A**) X-ray 2D projection and respective (**B**) 3D reconstruction of acquired structures in which the first column shows a reconstruction of both polymeric and ceramic phases, and the second column shows the reconstruction of the ceramic phase. A homogeneous distribution of the materials is observed, according to a colour scale: blue—soft material (mCol); brown—hard material (mBAp).

To address the morphological features of the composite scaffolds, microcomputed tomography (microCT) analysis was performed. A representative example of the 3D composite scaffolds was selected and shown in Figure 3 according to a colour scale, which was a result of a threshold applied for the different material densities (considering soft and hard densities for collagen and ceramic, respectively). A homogenous distribution of mBAp (brown colour) was observed within the collagen matrix (blue colour) (Figure 3), which suggests an optimized method of scaffolds production, characterized by a controlled freezing process. Quantitative information of porosity, pore size and interconnectivity was also obtained from microCT analysis, by modelling the structure with CTAn software, as depicted in the results outlined in Table 3. In general, for higher concentration of EDC/NHS and HMDI, 25% and 5%, respectively, a decrease of pore size, porosity and interconnectivity of pores was observed (Table 3). These results are in accordance with the SEM observation (Figure 2), which revealed a collagen mesh shrinkage for the higher EDC/NHS and HMDI crosslinker concentrations. This can be explained by a decrease in the number of free amino groups when the crosslinkers concentration increases as a result of a more extended crosslinking reaction [28,29]. Trabecular thickness for EDC/NHS conditions is larger as the crosslinker concentration increased, which was determined by modelling a filling of spheres in the structure by CtAn software. The same modelling system was used to calculate the interconnectivity, which decreases with the increase of the crosslinking agent concentration, as described.

Comparing the internal structure of EDC/NHS and HMDI crosslinked scaffolds, in general microCT results revealed higher pore size, porosity and interconnectivity for the latter ones. This result suggests a higher efficiency of EDC/NHS to crosslink collagen solutions, leading to the formation of a more compact structure. The reduced crosslinking effect of HMDI may be due to the unspecific reaction of isocyanates, which react not only with amine groups but also with hydroxyl groups (OH), when combined in liquid solutions. This result reveals the importance of using HMDI crosslink reaction under anhydrous conditions in order to reduce the reactivity of isocyanate with unspecific hydroxyl groups [25,30]. Taking into account the microCT and stability results, the efficacy of the scaffolds crosslinked with EDC/NHS at 25% and HMDI at 1% seems to be compromised.

In this way, the formulations of 5% HMDI and 12.5% EDC/NHS were chosen to carry on the study. Assuming a minimum pore size close to 100 µm to promote bone cell adhesion, migration and proliferation, the range of values obtained for both crosslinking conditions seems to be promising for cellular studies [31]. Moreover, obtained porosity (around 80%) comprises bone range porosity.

### 2.4. Composite Scaffolds’ Bioactivity

**Figure 4:**
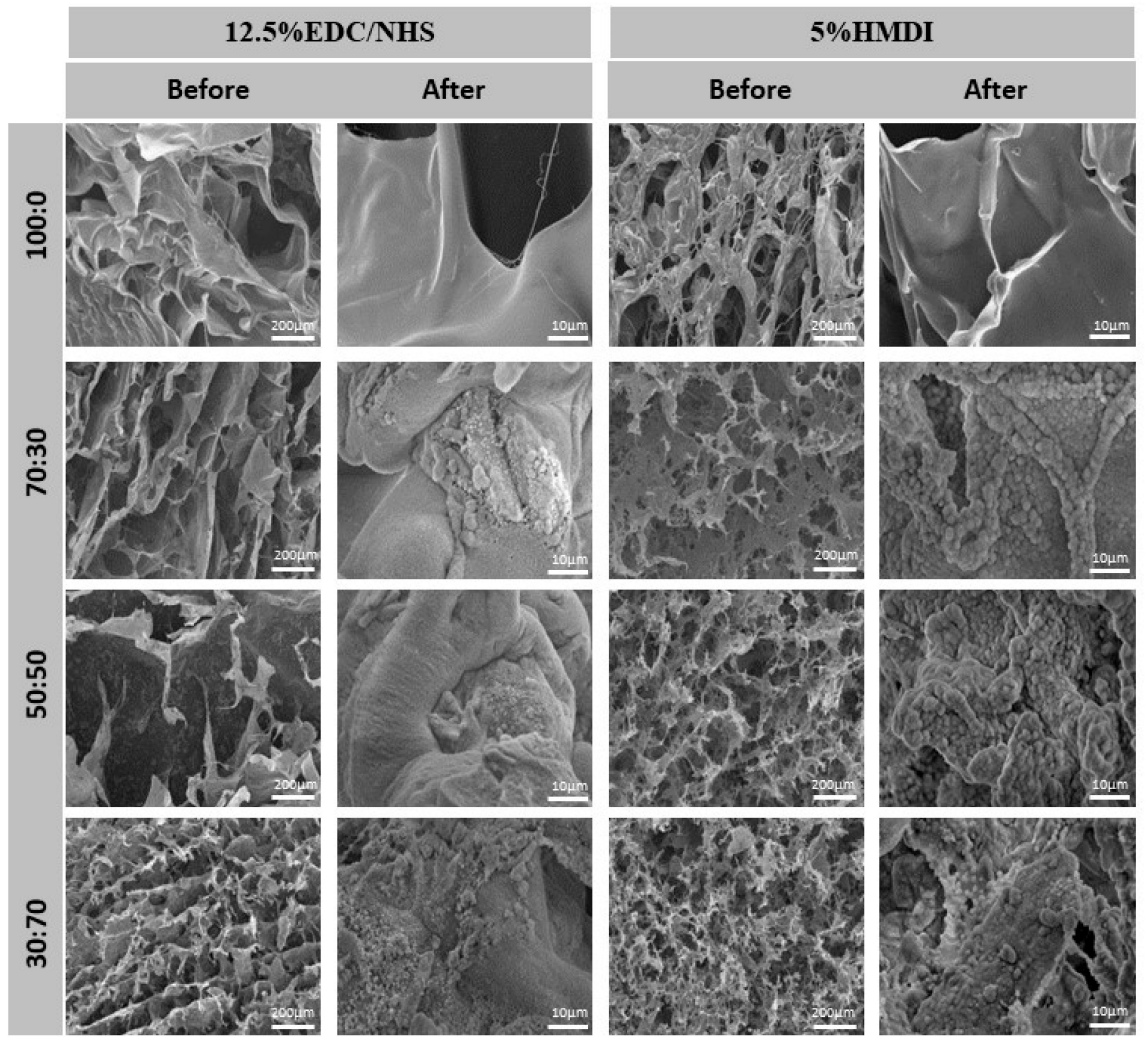
Representative scanning electron microscope (SEM) images of the mCol:mBAp composites scaffolds before and 14 days after the simulated body fluid (SBF) test confirming the bioactive nature of the formulations as shown by the presence of mineralized deposits.

The SEM images of the composite scaffolds 14 days after incubation in simulated Body Fluid (SBF) reveal the bioactive nature, i.e., the capacity to induce the mineralization process of the mCol:mBAp formulations’ structures. Comparing the images before and after SBF (Figure 4), it was possible to see that there was no mineralization in the collagen structures. In opposition, apatite deposition occurs in the mCol:mBAp structures and preferentially over the mBAp granules with the characteristic cauliflower structure. This is in agreement with other studies reporting the difficulty of bone-like apatite formation through the collagen matrix due to the absence of nucleation sites for apatite growth, highlighting the relevance of anionic groups as nucleating agents to promote apatite formation [32,33]. Indeed it is well known that the presence of calcium phosphates plays an important function in the mineralization process [30,34] and phosphate groups can attract calcium ions by electrostatic forces contributing to apatite formation [32]. In that sense, we can suggest particular interest in using the combination of mCol with mBAp to induce the mineralization process.

### 2.5. Mechanical Properties of Composite Scaffolds

**Figure 5:**
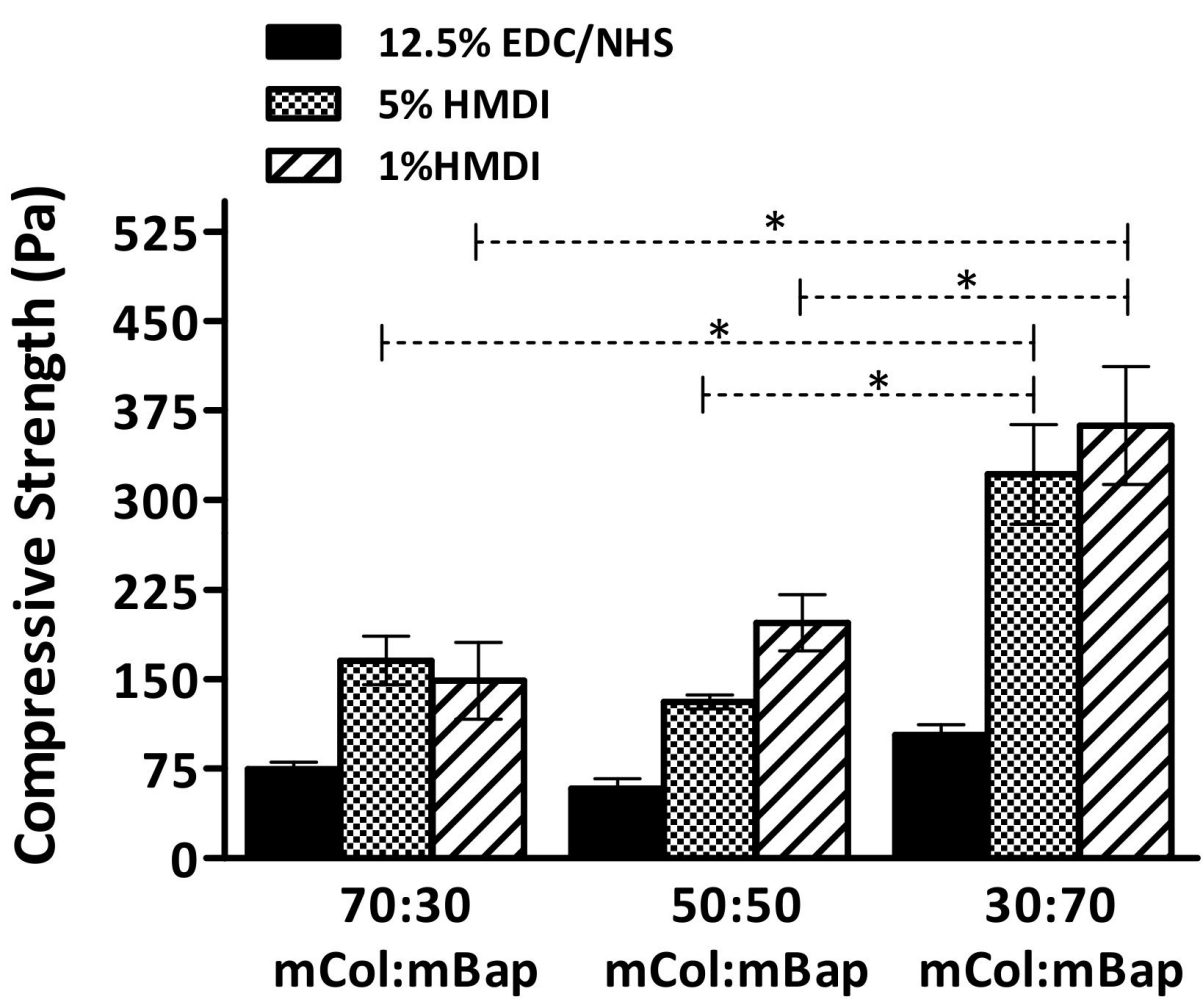
Compressive modulus of mCol:mBAp scaffolds crosslinked under different conditions. The Kuskal–Wallis test with Dunn’s multiple comparison test with a p value lower than 0.05 (* *p* < 0.05) was considered statistically significant.

**Figure 6:**
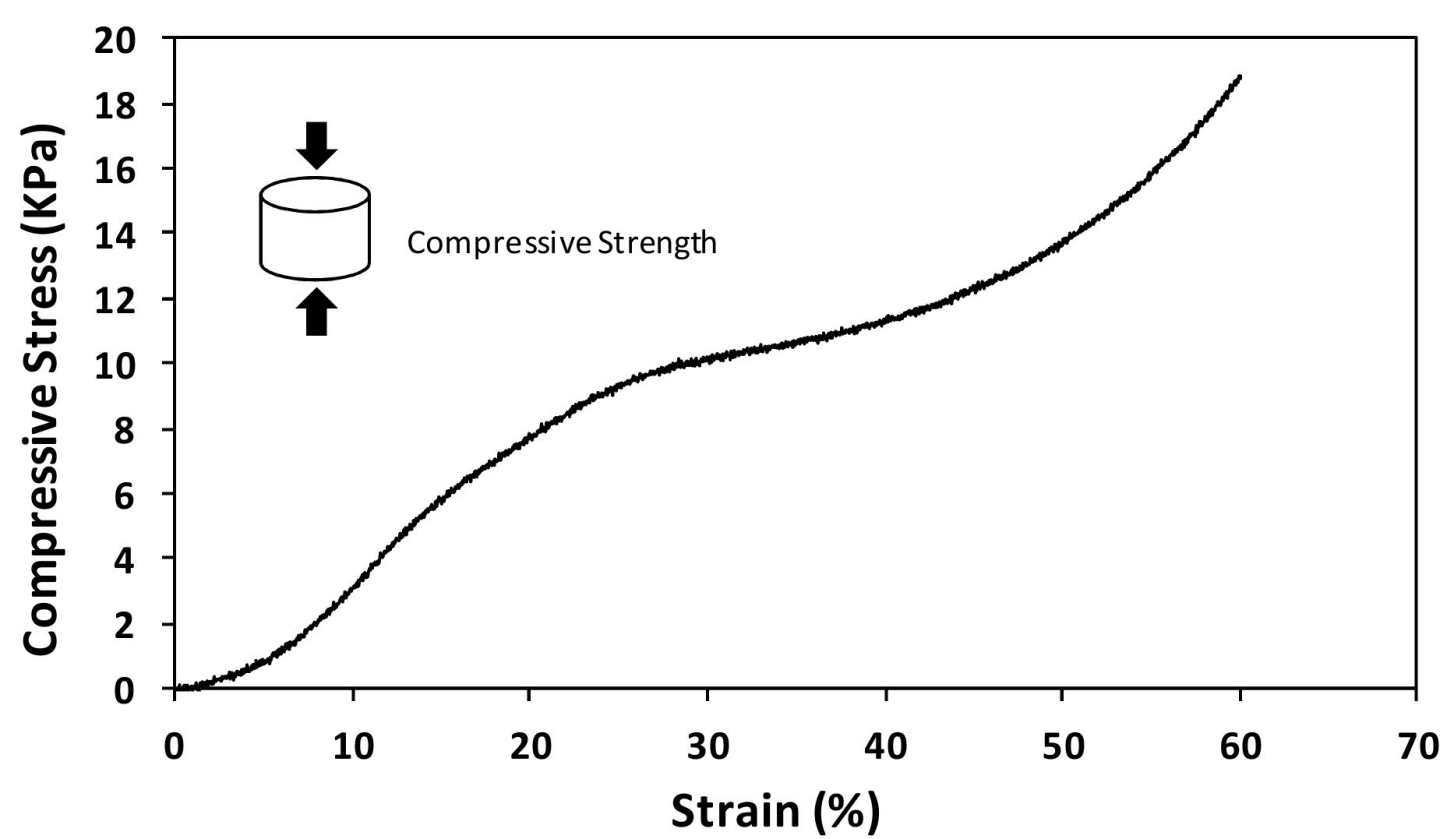
Representative example of a stress–strain curve for the mCol:mBAp composite scaffolds.

The mechanical properties of the developed composite scaffolds were characterized under a uniaxial compression load by evaluating the compressive elastic modulus. In general, the compressive modulus results (Figure 5) showed that the presence of mBAp significantly (*p* < 0.05) and successively improves scaffolds’ mechanical properties. Interestingly, the effect of bioapatite reinforcement was more pronounced in the scaffolds crosslinked with HMDI, but independently of the improvements the compressive modulus results are considerably lower than those of the trabecular human bone (100–000 MPa) [35]. Despite the poor mechanical properties of the scaffolds when compared with the native human bone, all scaffolds present stress–strain curves characteristic of highly ductile materials. In Figure 6, it is possible to see a representative example of the scaffolds’ mechanical behaviour, characteristic of flexible foams. This result suggests a high potential of such composites for surgical handling and fixation to the site of the defect.

### 2.6. Cell Viability in the Composite Scaffolds

In order to guarantee that the processing method did not result in any harmful residual compound, the metabolic activity of the osteoblast-like cell line (Saos-2) cultured in the scaffolds for 3 days was assessed.

**Figure 7:**
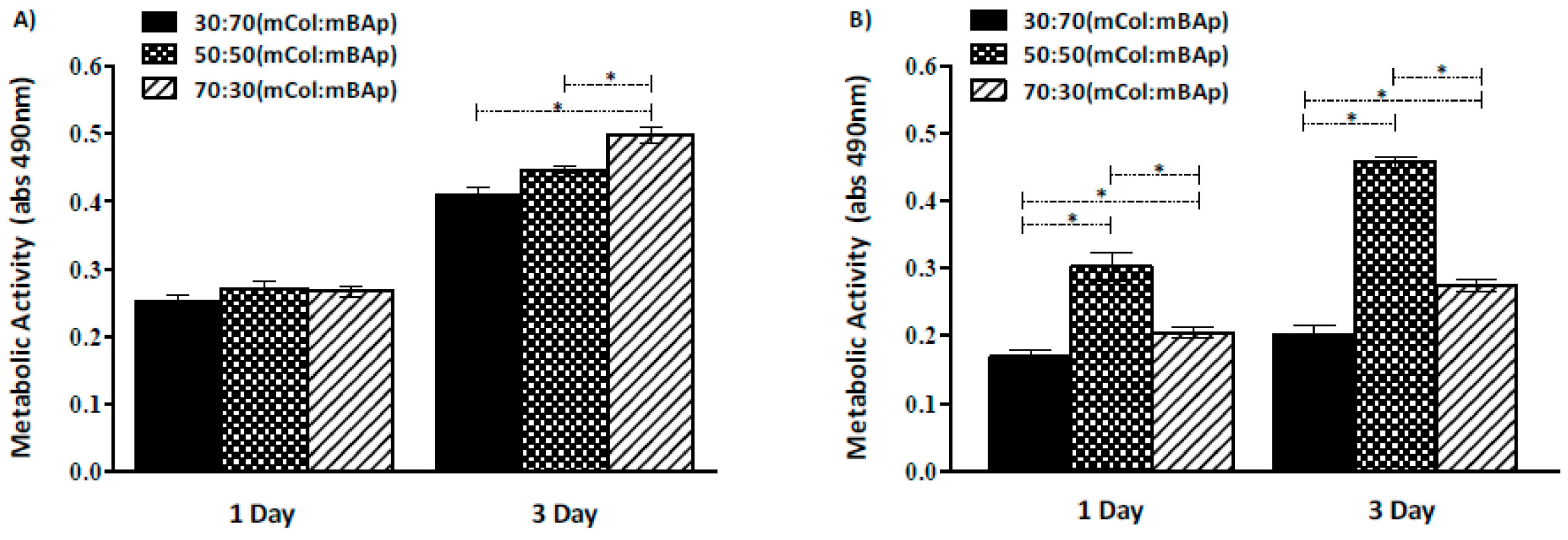
Metabolic activity of Saos-2 cells cultured in contact with mCol:mBAp scaffolds crosslinked with (**A**) 12.5% EDC/NHS and (**B**) 5% HMDI. Results are the mean ± standard error of three independent experiments. Statistical analysis was performed using the Kuskal–Wallis test with Dunns multiple comparison test (* *p* < 0.05).

Comparing the two graphs of the in vitro metabolic activity analysis (Figure 7), we can observe better results for the 12.5% EDC/NHS crosslinked scaffolds suggesting a higher cytotoxic effect of HMDI crosslinker. Similar results were achieved within the different mCol:mBAp EDC/NHS formulations, while for the HMDI crosslinked structures higher variability among the different formulations was observed.

**Figure 8:**
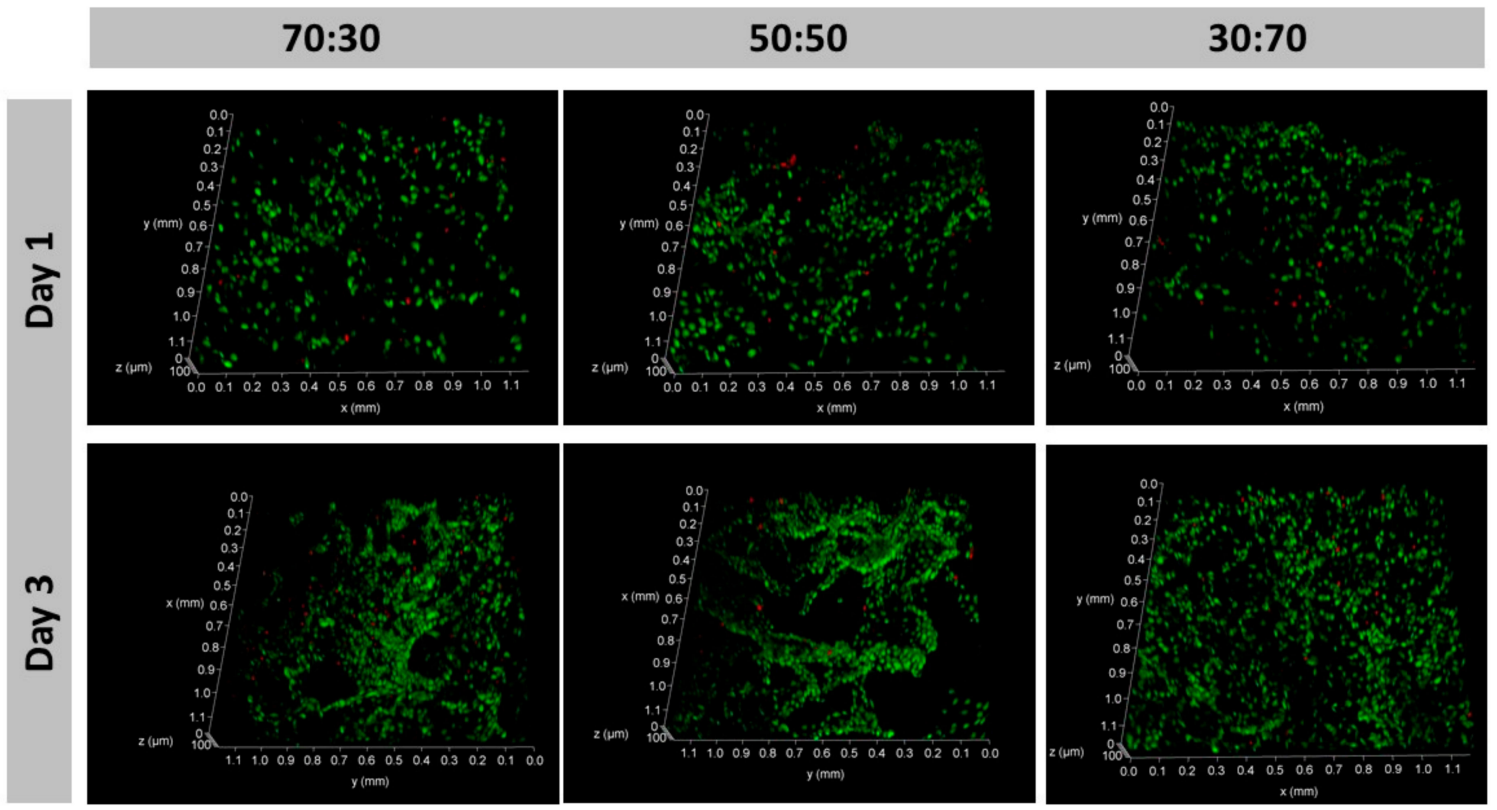
Saos-2 cell line adhered on 12.5% EDC/NHS composites 24 h after seeding and remains viable after 72 h. The majority of the cells are viable (green) as demonstrated by the abundant calcein (AM) stained cells in relation to the propidium iodide (PI) stained dead cells (red).

Considering both the structural stability and the metabolic activity results, the composite scaffolds crosslinked with 12.5% EDC/NHS were selected to further proceed with the biological performance assessment. Live/dead assay was then carried out to complement the MTS assay aiming to confirm the viability of the cells within the 3D structures and along the culture. Confocal laser scanning microscopy (CLSM) analysis showed the ability of the scaffolds to support an osteoblast-like cell culture as demonstrated by the viability of the majority of the cells that are within the structures up to 3 days of culture (Figure 8).

Such results confirm the positive effects of the bioapatite presence on cell biological activity already demonstrated in previous works [36,37,38]. In that sense, fish species can be used as a cheap source to obtain natural calcium phosphates with excellent biological properties.

From this, the selected composite scaffolds exhibited appropriate properties to be further considered for bone regeneration strategies, namely to consider their combination with stem cells promoting differentiation towards the osteogenic lineage.

## 3. Materials and Methods

### 3.1. Marine Collagen Extraction

Marine collagen (mCol) was extracted from shark skin (*Prionace glauca*) by the Instituto de Investigaciones Marinas (CSIC, Vigo, Spain). About 10 g of skin pieces were first treated with 0.1 N NaOH (1:10 *w*/*v*) by continuous stirring for 24 h at 4 °C to remove any non-collagenous protein present in the skin. Then, the liquid was discarded, and the remaining part was washed several times to stabilize the pH to 7. The resulting treated skin pieces were further stirred overnight with 10 volumes of 0.5 M acetic acid for the extraction of collagen. The extract was centrifuged at 10.000 g for 20 min at 10 °C. Supernatant was dialyzed withD9402-100FT dialysis membranes from Sigma with a molecular weight cut-off of 14,000 Da, using cold distilled water with several changes for 24–72 hand freeze-dried. This mCol was characterized as type I collagen by sodium dodecyl sulfate polyacrylamide gel electrophoresis (SDS-PAGE) as previously described by Sotelo et al. [39].

### 3.2. Marine Bioapatite Isolation

Marine bioapatite (mBAp) was obtained from shark teeth of *Isurus oxyrinchus* and *Prionace glauca*, provided by the Centro Tecnológico del Mar (CETMAR) and by the fishing company COPEMAR SA (Porto de Vigo, Spain), respectively. Teeth were cleaned and washed and further dried at 60 °C for 24 h. After this, teeth were ground in a ball mill (Retsch MM2000) for 5 min with an oscillation frequency between 90 to 100 Hz for powder of 100 to 0.1 µm. This powder of teeth was then pyrolyzed at 950 °C for 12 h with a heating ramp of 2 °C/min and a cooling ramp of 20 °C/min to remove the organic material of the tooth. The resulting granules of apatite were separated by sieves into a particle size between 20 to 63 µm.

### 3.3. Physicochemical Characterization

To evaluate the elementary composition of mBAp particles, ICP-OES and ion chromatography (only measuring fluorine) was carried out (Table 1). Four measurements per condition were taken and data are represented as mean value in weight percentage (wt %) and with standard deviation.

### 3.4. Scaffold Production

The 3D composites were produced by an optimized method of freeze-drying. Marine collagen was combined with marine bioapatite granules using different formulations: mCol:mBAp ratios of 100:0, 70:30, 50:50, 30:70. The freeze-dried collagen was solubilized at a final concentration of 1.5% in 0.5 M acetic acid followed by dialysis to ensure complete removal of impurities. After that, the isolated mBAp granules were added in the desired proportion to produce the aforementioned formulations. EDC/NHS or HMDI, the crosslinking agents selected for the present study, were added to the mCol:mBAp mixture at 12.5% or 25% for EDC/NHS and 1% or 5% for HMDI. After allowing the crosslinker to react (24 h), solutions with a viscous appearance were then poured into a mold and freeze-dried for at least 5 days. To wash out any harmful residual compound all scaffolds were thoroughly rinsed with distilled water immediately before using in cell culture experiments.

### 3.5. Stability Assay of 3D Structures

All the characterization studies were performed after sterilization with γ-irradiation (15 kGγ) [40]. The study of the scaffolds’ stability was carried out by incubation in culture medium during 14 days at 37 °C. During this period, samples were monitored and photographed to classify them according to their degree of stability. Samples that degraded 24 h after contact with culture medium at 37 °C were classify as unstable (−). Samples that degrades 7 days after incubation were reported as averagely stable structures (+) and the ones that maintains their integrity 14 days after culture medium contact were classify as high stable structures, signal (++).

### 3.6. Microcomputed Tomography of 3D Structures

Marine bioapatite particles distribution through the collagen matrix was assessed by microCT in terms of porosity (%), pore size (µm), trabecular thickness (µm) and pore interconnectivity (%, considering a minimum pore size of 58 µm). For scanning scaffolds (*n* = 3) a high-resolution microCT Skyscan 1072 scanner (Skyscan, Kontich, Belgium) with a voltage range and current source between 32–40 kV and 220–248 uA, respectively, was used. Quantitative parameters were measured by CT-Analyzer software (version 1.12.0.0) from 3D model reconstruction of a representative selection of slices and a designed region of interest. Thresholds between 30–60 and 120–255 was applied for the differentiation of soft (mCol) and hard (mBAp particles) materials, respectively. 3D images were finally obtained by CTvox software resulting in a colour scale directly related to the chosen threshold according to the material density/hardness.

### 3.7. Incubation in Simulated Body Fluid (SBS)

To understand the bioactive nature of the composites, SBF with an ion concentration of Na^+^ 142.0, K^+^ 5.0, Mg^2+^ 1.5, Ca^2+^ 2.5, Cl^−^ 147.8, HCO_3_^−^ 4.2, HPO_4_^3−^ 1.0 and SO_4_^2−^ 0.5 mM, similar to the concentration of human blood plasma (Na^+^ 142.0, K^+^ 5.0, Mg^2+^ 1.5, Ca^2+^ 2.5, Cl^−^ 103.0, HCO_3_^−^ 27, HPO_4_^2−^ 1.0 and SO_4_^2−^ 0.5 mM), was prepared according to Kokubo et al., 2006 [41]. Firstly, the dimensions of the previously produced composites were taken and the volume of SBF needed to submerse and characterize the bioactivity of those composites was determined through the following equation:

$$\mathrm{Vs}=\frac{\mathrm{Sa}}{10}$$

where Vs is the volume of SBF and Sa correspond to the apparent surface area of each specimen. For the produced scaffolds, the added volume of SBF was greater than that calculated (Vs), since they are porous structures. Three replicates of each condition were immersed in the SBF solution at 37 °C and pH 7.4 (mimicking the physiological conditions) for 1, 3, 7 and 14 days. After that, the samples were removed from the solution and washed with distilled water to avoid the formation of salts during the drying process. To evaluate apatite formation, samples before and after soaking in SBF were coated with gold and analysed by scanning electron microscopy (SEM) (JSM-6010LV, JEOL, Tokyo, Japan) with an acceleration voltage of 15 kV at different magnifications.

### 3.8. Mechanical Tests of 3D Structures

In order to study the mechanical behaviour of the mCol:mBAp structures, mechanical assays were carried out under compression mode using an INSTRON 5540. Five replicates of each condition were tested with a crosshead speed of 2 mm/min and a load cell of 1 kN. The compressive modulus, a measure of material stiffness was determined from the slope of the linear region of the stress-strain curve. However, for the formulations crosslinked with 25% of EDC/NHS, this assay was not determined since they did not present a uniform shape.

### 3.9. Evaluation of Cell Viability

Aiming at evaluating a potential cytotoxicity effect of the composites, Saos-2 cells were cultured in the 3D structures. Saos-2 cells were expanded in Dulbecco’s modified Eagle’s medium (DMEM) supplemented with 10% foetal bovine serum (ALFAGENE, Carcavelos, Portugal) and a 1% antibiotic–antimycotic (ALFAGENE) mixture. Cells were seeded on top of the different mCol:mBAp combinations in 48-well plates at a density of 2.5 × 10^4^ cells per scaffold. Metabolic activity at 1 and 3 days after seeding was determined using a 3-(4,5-dimethylthiazol-2-yl)-5-(3 carboxymethoxyphenyl) 2-(4-sulphofenyl)-2*H*-tetrazolium, inner salt (MTS) (VWR) assay. This assay quantifies the metabolic activity of the cells by the reduction of tetrazolium salt reagent to formazan after 3 h of incubation at 37 °C. Absorbance intensity, which is directly proportional to metabolic activity, was measured at 492 nm using a microplate reader (SYNERGY HT, BIO-TEK, Winooski, VT, USA). The results are the mean of three independent experiments (*n* = 3) with 3 replicates for each condition and per experiment [42].

Cell viability in the 12.5% EDC/NHS crosslinked scaffolds, which showed higher values of metabolic activity, was also confirmed by staining live/dead cells with calcein (AM)/propidium iodide (PI) (ALFAGENE), respectively. Briefly, Saos-2 cells were seeded in mCol:mBAp scaffolds, and 1 and 3 days after seeding, the culture medium was removed and replaced by a AM (1:1200) and PI (1:300) solution in culture medium. After 30 min of incubation in the dark, samples were washed twice with PBS and immediately visualized by CLSM (TCS SP8, Leica, Wetzlar, Germany). The acquired confocal images are the scaffolds’ mid-section. The scaffolds were cut in half and analysed according to the chosen parameters: slice thickness of 3.66 µm, depth of scanning of approximately 120 µm, and no sequential scanning. Regarding the latter parameter, before each experiment, acquisition conditions were tested with samples stained with only PI or only AM to ensure no crosstalk would be detected in the wrong channels.

### 3.10. Statistical Analysis

Statistical analysis of the results obtained for the different groups of scaffolds at varied conditions was performed by using Graph Prism Software. Data are presented as the mean ± standard deviation of a least three independent assays. First, a Shapiro–Wilk test was used to establish the assumption of data normality. Since data did not follow a normal distribution, a non-parametric test was used (Kruskal–Wallis test, with Dunn’s multiple comparison test and A p value less than 0.05 (* *p* < 0.05) was considered statistically significant.

## 4. Conclusions

Marine organisms emerge as an attractive alternative to mammalian ones as raw materials for the production of biomaterials despite the challenge posed by processing high-quality and low-cost bioactive compounds from fish waste.

The feasibility of fabricating collagen/apatite scaffolds from by-products of marine resources, with a homogeneous distribution of apatite particles throughout the collagen matrix, was successfully accomplished in this work. Despite the scaffolds’ properties being affected by different parameters, such as the mCol:mBAp ratio and the crosslinker agent, bioactive stable composite structures were developed. Marine-origin col:BAp scaffolds crosslinked with 12.5% EDC/NHS appear to be suitable as potential structures for bone regeneration therapeutic approaches. Ongoing work in our lab is underway to examine their potential to promote osteogenic differentiation.

## Figures and Tables

**Table 1:** Elemental composition (% of weight) of bioapatite determined by inductively coupled plasma optical emission spectrometry (ICP-OES) and ion chromatography analysis.

Element	Weight (%)
Ca	44.364 ± 5
P	22.8 ± 2.3
F	1.0 ± 0.5
Na	0.9 ± 0.2
Mg	0.65 ± 0.04
Sr	0.25 ± 0.02
K	0.018 ± 0.002
Al	0.007 ± 0.005
Fe	0.006 ± 0.003

**Table 2:** Stability of the produced scaffolds after 14 days in culture medium at 37 °C. (−) represent the structures that were completely degraded 1 day after culture medium, (+) structures that were completely degraded 7 days after incubation (++) structures that maintained integrity 14 days after culture incubation.

mCol:mBAp	25% EDC/NHS	12.5% EDC/NHS	1% HMDI	5% HMDI
100:0	+	−	−	−
70:30	++	++	−	++
50:50	++	++	−	++
30:70	++	++	−	++

**Table 3:** Microarchitecture features of the different mCol:mBAp composite scaffolds determined by microCT analysis.

mCol:mBAp	Crosslinker	Mean Pore Size (µm)	Porosity (%)	Trabecular Thickness (µm)	Inter-Connectivity (%)
100:0	25% EDC:NHS	45.5 ± 11.7	48.8 ± 14.8	45.1 ± 8.7	46.4 ± 15.9
100:0	12.5% EDC:NHS	64.1 ± 5.7	72.7 ± 1.8	35.4 ± 5.4	69.3 ± 7.7
100:0	5% HMDI	52.7 ± 11.0	76.3 ± 4.2	27.9 ± 5.5	81.5 ± 8.5
100:0	1% HMDI	115.1 ± 35.6	87.2 ± 1.3	33.2 ± 2.9	69.4 ± 15.1
70:30	25% EDC:NHS	56.5 ± 10.7	48.9 ± 3.4	51.9 ± 4.6	54.3 ± 1.9
70:30	12.5% EDC:NHS	83.4 ± 11.8	78.3 ± 5.7	39.4 ± 1.6	92.1 ± 3.0
70:30	5% HMDI	147.0 ± 38.4	90.4 ± 3.2	41.6 ± 2.7	97.3 ± 1.8
70:30	1% HMDI	161.4 ± 13.7	91.8 ± 2.0	44.4 ± 3.2	97.4 ± 2.1
50:50	25% EDC:NHS	50.5 ± 1.6	49.0 ± 3.1	47.9 ± 2.3	49.8 ± 4.8
50:50	12.5% EDC:NHS	126.3 ± 22.5	85.4 ± 3.2	43.3 ± 0.67	96.8 ± 1.8
50:50	5% HMDI	142.6 + 24.1	89.4 ± 3.0	43.2 ± 2.2	97.4 ± 1.6
50:50	1% HMDI	155.1 ± 31.3	87.8 ± 5.3	46.1 ± 4.6	87.0 ± 11.8
30:70	25% EDC:NHS	113.8 ± 16.0	69.3 ± 1.3	52.8 ± 2.1	65.7 ± 7.9
30:70	12.5% EDC:NHS	104.4 ± 15.7	73.9 ± 1.9	47.0 ± 1.2	85.4 ± 2.4
30:70	5% HMDI	62.6 ± 6.8	67.5 ± 4.2	40.8 ± 1.7	68.8 ± 9.8
30:70	1% HMDI	172.0 ± 43.3	86.8 ± 8.2	46.8 ± 6.0	89.5 ± 9.5
